# Semiconductor quantum dots for photodynamic therapy: Recent advances

**DOI:** 10.3389/fchem.2022.946574

**Published:** 2022-08-11

**Authors:** Bhawna Uprety, Heidi Abrahamse

**Affiliations:** Laser Research Centre, Faculty of Health Sciences, University of Johannesburg, Johannesburg, South Africa

**Keywords:** cancer, nanotechnology, quantum dots, photosensitizers, photodynamic therapy

## Abstract

Photodynamic therapy is a promising cancer treatment that induces apoptosis as a result of the interactions between light and a photosensitizing drug. Lately, the emergence of biocompatible nanoparticles has revolutionized the prospects of photodynamic therapy (PDT) in clinical trials. Consequently, a lot of research is now being focused on developing non-toxic, biocompatible nanoparticle-based photosensitizers for effective cancer treatments using PDT. In this regard, semiconducting quantum dots have shown encouraging results. Quantum dots are artificial semiconducting nanocrystals with distinct chemical and physical properties. Their optical properties can be fine-tuned by varying their size, which usually ranges from 1 to 10 nm. They present many advantages over conventional photosensitizers, mainly their emission properties can be manipulated within the near IR region as opposed to the visible region by the former. Consequently, low intensity light can be used to penetrate deeper tissues owing to low scattering in the near IR region. Recently, successful reports on imaging and PDT of cancer using carbon (carbon, graphene based) and metallic (Cd based) based quantum dots are promising. This review aims to summarize the development and the status quo of quantum dots for cancer treatment.

## GRAPHICAL ABSTRACT

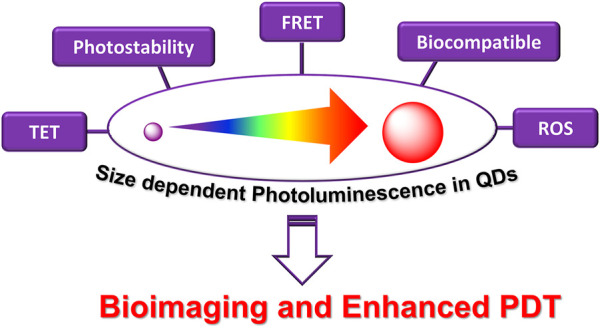

## Introduction

### Photodynamic therapy and cancer

**FIGURE 1:**
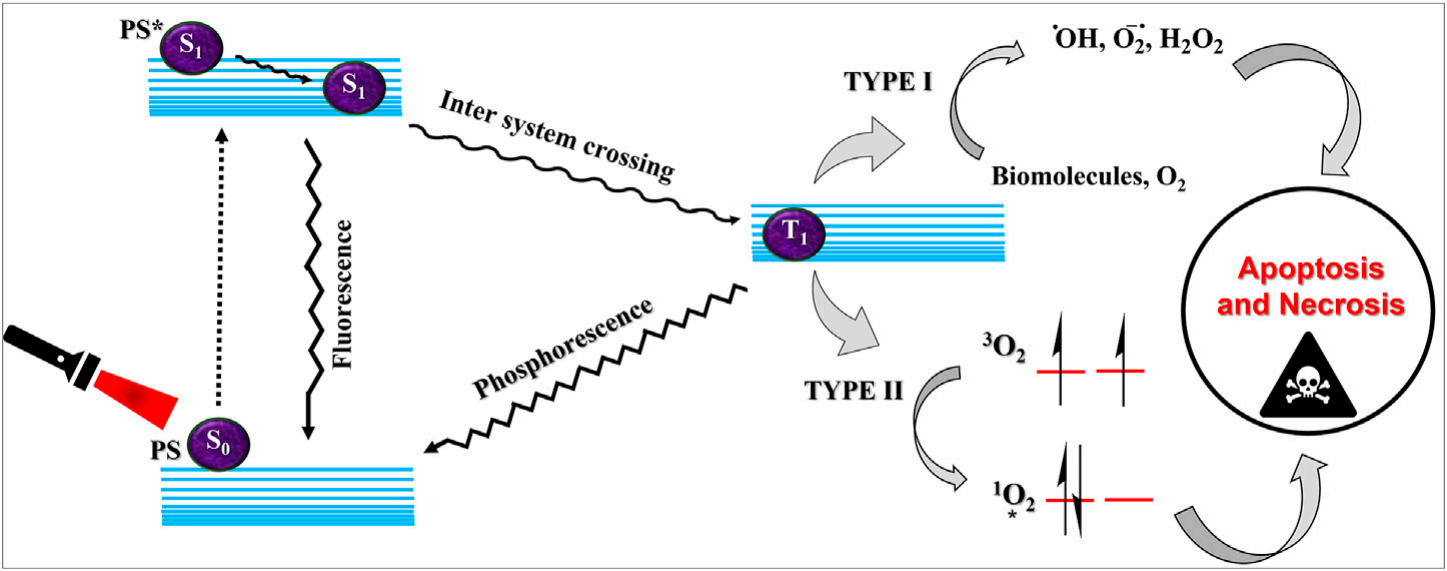
Schematic explanation of photodynamic therapy. The ground state photosensitizer (S0) on suitable irradiation, gets excited to the singlet state (S1). This singlet state PS can fluoresce back to the ground state or can convert to the triplet excited state (T1) via intersystem crossing. The triplet state can transfer energy via type I or type II processes leading to the generation of reactive oxygen species and execution of photodynamic therapeutic action.

Cancer is a dreadful disease and the leading cause of mortalities in developed as well as developing countries. As per the global cancer statistics, a record of 10 million cancer deaths were reported in 2020 (Sung et al., 2021). Therefore, cancer biology has been researched extensively to understand the mechanisms of cancer growth and metastasis, and subsequently, to develop better, effective, and safer treatment modalities. Accordingly, several novel approaches are being used clinically, or are in clinical trials, including combination chemotherapy (Chung et al., 2020; Yamada et al., 2022), immunotherapy (Kraehenbuehl et al., 2022; Li et al., 2022b), radiation (Tchelebi et al., 2020; McGovern et al., 2022), and photodynamic therapy (Li et al., 2020a; Lo et al., 2020). Although cancer research has advanced progressively, there are only a few notable treatment improvements (Agostinis et al., 2011). Furthermore, most of the cancer drugs and treatments are often associated with severe side effects due to the systemic circulation of the drug molecules (Partridge et al., 2001; Kayl and Meyers, 2006; Aslam et al., 2014). Recently, photodynamic therapy (PDT) has garnered increasing attention in cancer research. PDT is a noninvasive targeted technique employing a photosensitizing drug molecule that gets activated upon irradiation of a light source (Brown et al., 2004; Robertson et al., 2009; Abrahamse and Tynga, 2018). The photosensitizer (PS) gets excited to the transient singlet excited state following photon absorption on light irradiation. The excited singlet state can follow two fates—it can either fluoresce back to the ground state, or it can relax to a relatively stable excited triplet state following intersystem crossing. The excited triplet state then relaxes back to the ground state transferring energy to molecular oxygen and thereby generating singlet oxygen species (^1^O_2_) (Type II process). Singlet oxygen is an extremely strong oxidizing agent which can readily oxidize biomolecules such as proteins, lipids, DNA, RNA thus killing cancer cells (Wilson, 2002; Robertson et al., 2009). Besides, the triplet state PS can lose a hydrogen to generate radicals and anion radicals that initiates the therapeutic effect (Figure 1) (MacRobert and Theodossiou, 2005). PDT can have varying efficacies and mechanisms of therapeutic action based on the kind of drug and dose, light source, tissue type and availability of oxygen (Woodhams et al., 2004). There are three generally accepted mechanisms of cell death triggered by PDT. 1) PDT may result in vascular constriction and platelet aggregation; 2) it may also induce apoptosis and necrosis via direct cellular oxidation, 3) and finally, it may initiate autoimmune and inflammatory responses (Juarranz et al., 2008). PDT offers several advantages over conventional cancer therapies. Most importantly, it does not require any surgical interventions and it can be localized at the tumor site with laser irradiation, thereby limiting systemic toxicity. The first clinical application of PDT dates back to the 1990s using hematoporphyrin derivatives and photofrin (II), and since then, several advancements have been made to develop better photosensitizers, light sources, as well as in the basic understanding of photochemistry and photobiology (Wilson, 2002).

Photosensitizers (PS) form an important part of photodynamic therapy. Most photosensitizers used in cancer research and therapy are based on porphyrin, chlorin, and phthalocyanine backbone (Abrahamse and Hamblin, 2017; Zhang et al., 2018). An ideal photosensitizer must be chemically pure and easy to synthesize with long shelf life. The quantum yield of the metastable triplet state should ideally approach unity, and the PS should have a strong absorption preferably between 600–800 nm to provide for singlet oxygen excitation energy (94 kJ/mol). Furthermore, it should exhibit minimal dark toxicity and must possess a high renal clearance rate. They must be able to selectively localize at the tumor tissue (Robertson et al., 2009; Abrahamse and Hamblin, 2017). The first generation photosensitizers were based on hematoporphyrin backbone and were potent against breast, colorectal, oral, brain and lung cancers. However, they suffered few major setbacks such as complicated synthesis and complex structures, low quantum yields, slow pharmacokinetics, hydrophobic nature, and poor tissue penetration due to short wavelengths (Park et al., 2021). The constraints of water solubility and stability were partially met by introducing few structural changes in the cyclic tetrapyrrole ring such as the introduction of sulphonyl groups. This led to the development of substituted phthalocyanines and chlorins (temoporfin, hexylpyropheophorbide) as the second generation photosensitizers. The second generation PS exhibit greater tissue selectivity as well and due to longer absorbance wavelengths, they can also be used to target deeper tissues (Josefsen and Boyle, 2008b; Kou et al., 2017). 5-Aminolevulinic acid is an example of a successful second generation photosensitizer. It acts as a precursor and gets metabolized to protoporphyrin IX post administration (Josefsen and Boyle, 2008b). Besides, few second generation PSs are designed to target specific cellular functions (Yuan et al., 2016; Yu et al., 2017), for example DLC-porphyrin conjugates that preferentially localize in the mitochondria (Kou et al., 2017). Additionally, few bio-conjugated photosensitizers have also been proposed for imaging and therapy of cancer (Van Dongen et al., 2004; Simões et al., 2020). A list of few photosensitizers in clinical trials has been included in Table 1. The third generation photosensitizers are the more advanced form of PS involving classical photosensitizers conjugated to specific proteins, amino acids, antibodies, carbohydrates for target specific action. They may also include metal organic framework based nanozymes (Huang et al., 2021), classical PS encapsulated or conjugated to drug delivery carriers such as nanoparticles, supramolecular assemblies for improved accumulation of PS at the tumour site thereby reducing systemic toxicity (Josefsen and Boyle, 2008a; Setaro et al., 2020; Mfouo-Tynga et al., 2021).

**TABLE 1:** List of few photosensitizers in clinical trials.

Structure	Photosensitizer (Trade name)	λ nm (ε M^−1^ cm^−1^)	Cancer type	Clinical trial status
First generation photosensitizers				
Hematoporphyrin				
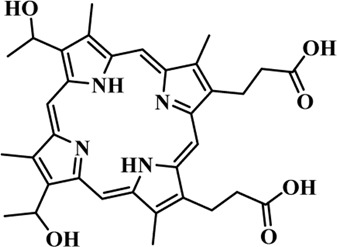	Porfimer sodium (Photofrin)	630 (3·0 × 10^3^)	Lung, esophagus, bladder, ovarian	Approved worldwide
Second Generation Photosensitizers				
Protoporphyrin prodrug				
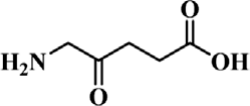	5-Aminolevulinic acid (5-ALA) (Levulan)	635 (<10^4^)	Skin, bladder, brain, esophagus	Approved worldwide
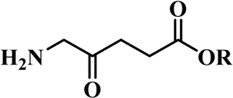	5-ALA-methylester (Metvixia)		Skin, bladder	US FDA, EU, New Zealand, Australia
	5-ALA-benzylester (Benzvix)		Gastrointestinal cancer	Not approved
Benzoporphyrin				
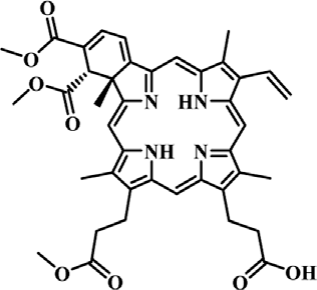	Verteporfin (Visudyne)	689–693 (3·5 × 10^4^)	Ophthalmic, pancreatic, skin	US FDA, EU, Canada
Chlorins				
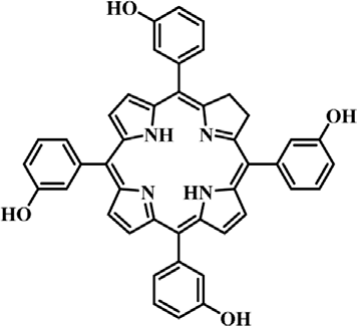	Temoporfin (Foscan)	652 (3·0 × 10^4^)	Head, neck, prostrate, pancreas	European Union
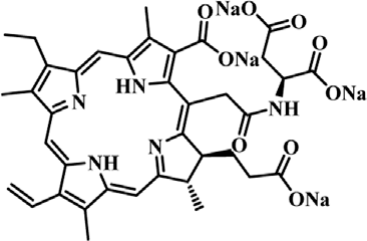	Talaporfin sodium (Aptocine/Laserphyrin)	664 (4·5 × 10^4^)	Lung cancer and solid tumors	Japan
Phthalocyanine				
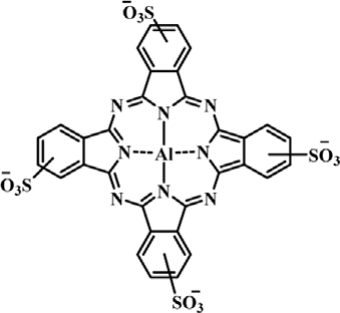	Sulfonated aluminium phthalocyanine (Photosens)	675 (1·1 × 10^5^)	Various cancers, AMD	Russia
Texafrins				
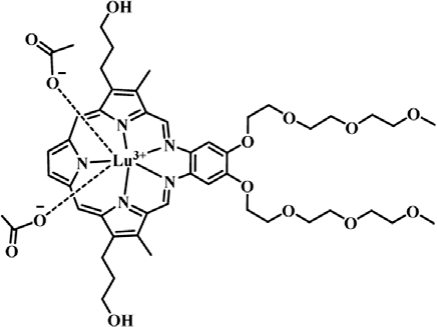	Motexafin lutetium (Antrin)	732 (4·2 × 10^4^)	Prostate cancer	Terminated
Pheophorbide-a				
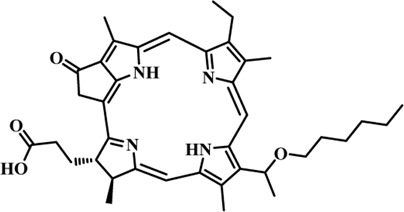	2-(1-Hexyloxyethyl)-2-devinyl pyropheophorbide-a (Photochlor)	665 nm (4·75 × 10^4^)	Lung cancer	Clinical trials
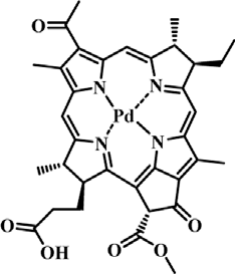	Palladium bacteriopheophorbide (Tookad/WST09)	763 (>10^5^)	Prostate cancer	
Purpurin				
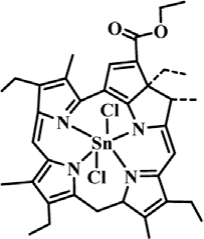	Rostaporfin (Photrex)	660 (2·8 × 10^4^)	Breast, basal cell carcinoma, prostate cancer	United States (Phase II)
Porphycene				
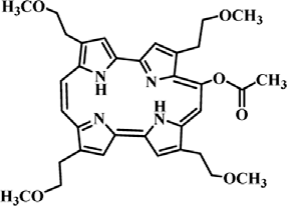	9-acetoxy-2,7,12,17-tetrakis-(β-methoxyethyl)-porphycene (ATMPn)	610–650 (5·0 × 10^4^)	Psoriasis, non melanoma skin cancer	Germany

### Current limitations of PDT

Despite the remarkable success of second generation PS, their widespread clinical applications are limited by the fact that most of them tend to accumulate in the skin and eyes leading to phototoxicity and photosensitivity for prolonged periods. Therefore, tumor selectivity is seldom achieved. Moreover, most photosensitizers have hydrophobic extended π networks, and thus tend to agglomerate in aqueous media. Lastly, widespread application of PDT as an independent treatment modality is also restricted by the fact that it is not very successful for deep rooted and bulky tumors, as well as for tumors that have metastasized to multiple organs (Lucky et al., 2015; Gunaydin et al., 2021). Therefore, the current research is focused on developing third generation photosensitizers which can overcome the drawbacks of solubility, and phototoxicity to normal cells. Extensive modifications in the PS structure such as conjugation with cellular targeting peptides and antigens have been assessed to allow for the targeted localization of PS at the tumor site (Wu, 2018; Simões et al., 2020). Nanotechnology has also been incorporated into PDT to allow for better conjugation and targeted delivery of the photosensitizers.

### Nanotechnology in PDT

The clinical success of Photofrin encouraged the development of PDT as a promising treatment modality for cancer. However, many potent photosensitizers are restrained for widespread clinical use for the reasons outlined above. Therefore, current research is being focused on combating these drawbacks and designing drug delivery carriers and/or stabilizers for PDT, and so far, nanotechnology has yielded promising results. Nanotechnology is an emerging tool, being used to overcome the existing limitations associated with photodynamic therapy and for the development of advanced third generation photosensitizers (Abrahamse et al., 2017; Chizenga and Abrahamse, 2020; Park et al., 2021). Moreover, nanoparticles can target tumors due to enhanced permeability and retention properties, leading to targeted photodynamic action, thereby limiting the phototoxicity to normal and healthy tissues (Hamblin et al., 2015; Sivasubramanian et al., 2019). Nanocarriers for photosensitizers for PDT can provide many advantages such as localization of the PS at the tumor site, delivery of high concentrations of PS, improved biodistribution by surface modifications and many more (Lucky et al., 2015; Qidwai et al., 2020; Sztandera et al., 2020). Consequently, many nano-PS conjugates have been prepared and reviewed for their PDT action (Patel et al., 2017; Park et al., 2018; Yang et al., 2018; Nkune et al., 2021). Additionally, few semiconductor nanoparticles also known as quantum dots are gaining emphasis for their ability to generate singlet oxygen, thereby eliminating the need of conventional PS. Moreover, some of these QDs have also been conjugated to classical PSs for enhanced photoluminescence and PDT action via two photon emission (TPE) and fluorescence energy transfer (FRET). This review is focused on reviewing the development and future of semiconducting quantum dots as photosensitizers for PDT.

## Semiconductor quantum dots

**FIGURE 2:**
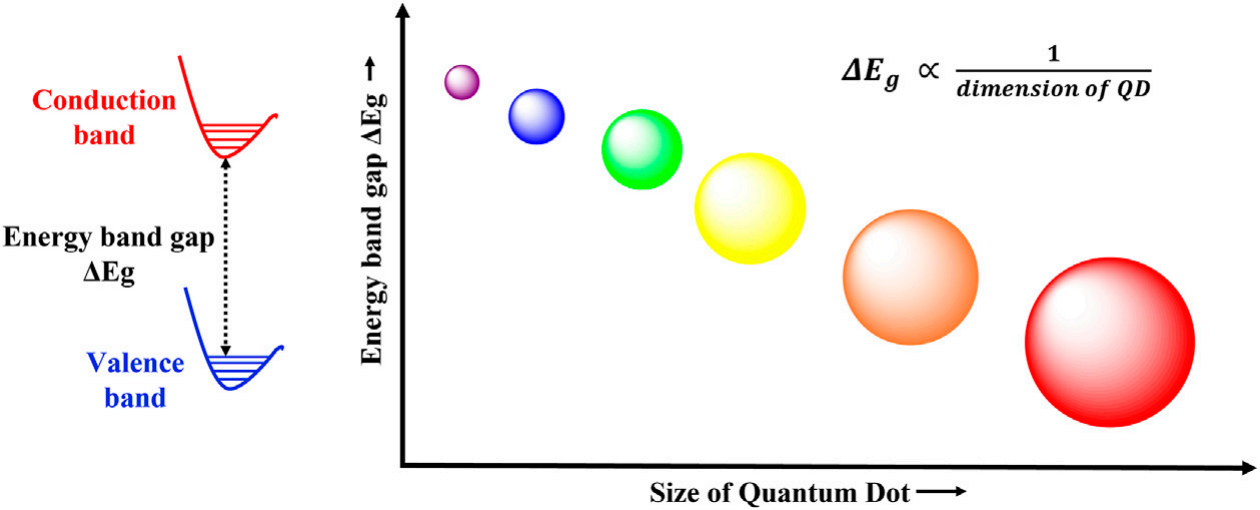
Size dependent photoluminescence in QDs. The band gap energy and the size of quantum dots follows an inverse relationship. Therefore, QDs with large band gap energies have smaller radii, while small band gap QDs are larger in size.

Nanomaterials display interesting and distinct properties as compared to bulk materials owing to their nano dimensions and the consequent electron confinement effects. Nanomaterials are usually classified based on the number of degrees of freedom experienced by the electrons and holes in them (Sumanth Kumar et al., 2018). Quantum wells are two dimensional nanomaterials, with confinement in one direction. Quantum wires are one dimensional nanostructure with confinement in two directions, and finally quantum dots are zero dimensional semiconductor nanocrystals with confinement in all three directions. Their size ranges from 1 to 10 nm, and they are composed of a few tens to thousands of atoms, with electrons quantized in all directions. Thus, the electronic energy levels in quantum dots (QDs) are discreet like in atoms or molecules, and hence QDs are also sometimes referred to as artificial atoms. They possess an intrinsic band gap that allows the excitation and bridging of electrons. This band gap is inversely related to the size of QD, greater the size, smaller the band gap (Efros and Brus, 2021). Thus, the QD band gap energy and hence their optoelectrical properties can be manipulated by varying their size (Hong, 2019), larger QDs have smaller band gaps while smaller QDs have large band gaps (Figure 2).

### Classification of quantum dots

Quantum dots can have varied compositions and can be classified into core type QDs, core shell QDs, and alloyed QDs based on their chemical structure (Liang et al., 2021). Core type QDs are composed of a single material, usually a metallic chalcogenide (CdS, CdSe, CdTe, PbS) and have homogeneous compositions. Metal free QDs such as silicon quantum dots and carbon based QDs such as carbon quantum dots and graphene quantum dots also fall in this category. They depict tremendous fluorescent properties and have been extensively explored for sensing and bioimaging applications (Farzin and Abdoos, 2021).

**FIGURE 3:**
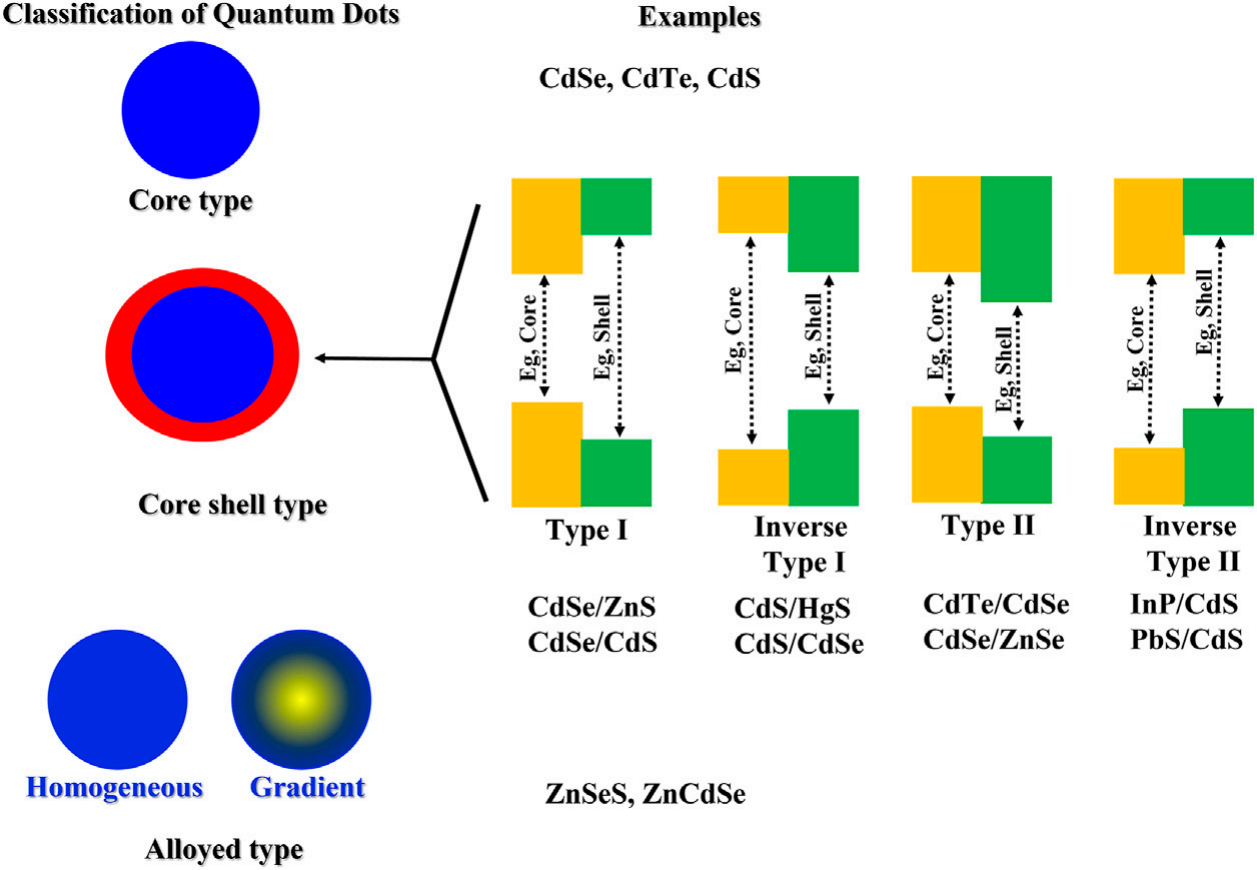
Classification of quantum dots with few examples of each type.

Core shell QDs are advanced quantum dots and are composed of a core type material encapsulated within a second semiconductor. The embedding of the quantum core within a shell of another semiconductor material prevents the nonradiative decay of the exciton, leading to enhanced photoluminescence as well as improved stability. Typical compositions employed for the core and shell are type II–VI, IV–VI, and III–V semiconductors (Cotta, 2020). Few examples include CdSe/Zns, CdSe/CdS, InAs/Cds. Depending upon the band gaps in the core and shell semiconductor materials, core shell type QDs can be further classified into type I, inverse type I, type II and inverse type II quantum dots (Figure 3). The biocompatibility of core shell QDs has also been improved by doping with transition metals (AbouElhamd et al., 2019). Finally, alloyed QDs are composed of two semiconductors with different band gap energies (examples: CdSeTe, CdSeS). Their composition follows a concentration gradient allowing the manipulation of optical properties without altering the particle size (Bailey and Nie, 2003).

### Synthesis of QDs

**FIGURE 4:**
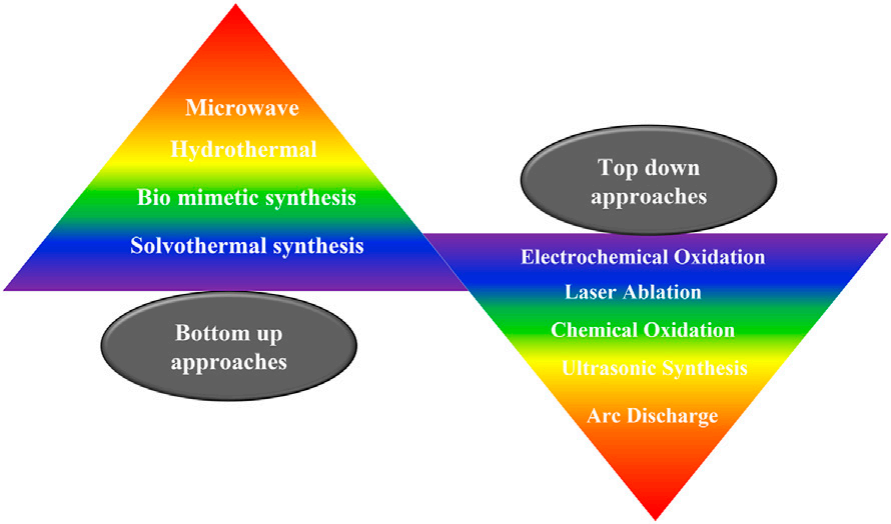
Common synthetic protocols for the synthesis of quantum dots. QDs can be synthesizes via top up or bottom down approaches.

Quantum dots can be synthesized by chemical, physical or biological processes, and the synthetic method followed plays an important role in the size and the consequent photoluminescence properties as well as the biocompatibility of the QDs (Zhou et al., 2015). Broadly, the approaches can be categorized as top down and bottom up syntheses. Top down synthetic routes involve the thinning or slicing of a bulk semiconductor to yield the quantum dot. These are mature strategies with abundant raw materials. Examples include focused ion or laser beam, electron beam lithography, wet or reactive ion etching. These methods are usually facile and ecofriendly; however, the resulting quantum dots often possess impurities, surface defects, have poor stability and low quantum yields. Bottom up approaches usually involve self-assembly and may further be classified into wet chemical and vapor phase methods. Bottom up approaches are usually inspired by biology; they provide precise control over size and have fewer defects. However, the synthetic protocols are often complex and complicated involving organic solvents. Bottom up approaches include hydrothermal methods, solvothermal methods and microwave synthesis. Few commonly employed synthetic protocols for the synthesis of QDs are presented in Figure 4. The synthetic methods for quantum dots have been reviewed in great detail by many researchers, and hence will not be elaborated here (Bera et al., 2010; Valizadeh et al., 2012; Karakoti et al., 2015; Wang et al., 2017; Pu et al., 2018; Singh et al., 2019).

### Quantum confinement effects

**FIGURE 5:**
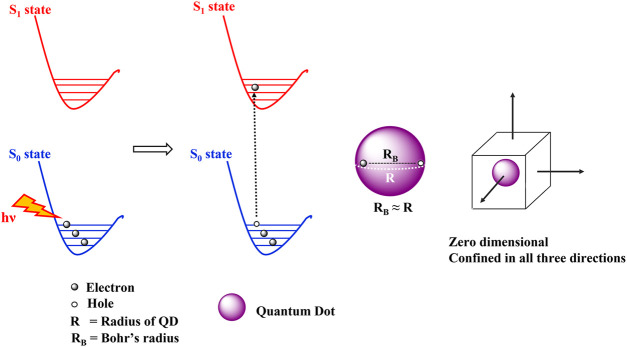
Quantum confinement effects seen in quantum dots. The Bohr’s radius of the exciton is similar to the radius of the QD leading to the spatial confinement of electrons in all the three directions.

The unique electronic and optical properties of QDs are attributed to the quantum confinement effects. The quantum confinement effects can be understood in terms of the Bohr radius which is defined as the distance between an electron in the conduction band and its corresponding hole in the valence band. The Bohr radius is characteristic of each semiconductor and quantum confinement effects are observed at dimensions smaller than the Bohr radius. The dimensions of a bulk semiconducting material are much greater than the Bohr radius and thus the electronic energy levels are continuous. A quantum dot on the other hand, is extremely minute comprising of only a few atoms. When quantum dots are excited by a photon of energy hν, the electrons in the valence band gains energy and forms the exciton. An exciton is an electron-hole pair held together by Columbic electrostatic forces. The size of this exciton is similar to the size of QD (Figure 5), leading to discreet atomic like energy levels and the spatial confinement of the exciton in three dimensions. The discreet atom like energy levels also result in longer lifetime of the excited states (Mandal and Chakrabarti, 2017; Zaini et al., 2020).

### Typical properties of quantum dots—tunable photoluminescence and band gap

The most widely appreciated property of quantum dots is their size dependent luminescence. QDs of similar materials, but different dimensions possess different colors (Figure 2). As explained in *Graphene quantum dots*, when a photon of sufficient energy strikes a QD, the electron from the valence band gets excited to the conduction band, thus forming a hole in the conduction band and the exciton. Next, the exciton relaxes back to the lower energy state by emitting some energy and recombing the electron and the hole in the process. The emitted energy is expressed as the sum of the bandgap energy, the quantum confinement energy, and the bond energy of exciton (Sumanth Kumar et al., 2018); and the process involves conventional fluorescence emission of a longer wavelength (red shifted) photon (Bentolila, 2015). Thus, as smaller QDs have large band gaps, more energy will be required to form the corresponding exciton. Hence greater energy wavelengths will be emitted during luminescence. Thus, quantum dots display size dependent luminescence properties. This becomes particularly crucial in designing tailor made QDs for specific luminescent applications.

Thus, the emission spectra of QDs can be easily modified to emit anywhere between the UV to near IR (NIR) region, as against the visible region with conventional photosensitizers, just by changing their size. The control over size of QDs can be achieved by varying the reaction parameters like pH, temperature, time, concentration, etc. during the synthetic pathway. They can also be tailored to have two photon cross section absorption, allowing excitation in the near infrared (NIR) range. Within the NIR window, the propagating light diffuses rapidly leading to deep tissue penetration. Thus QDs can be employed with low intensity lights to target deep rooted tumors (Samia et al., 2003). They can also be used for bioimaging applications. As compared to conventional organic dyes, they can be excited by a broad range of irradiation wavelength (they possess broad absorption spectra) but exhibit narrow and sharp fluorescence emission peaks. They have high extinction coefficients, indicating their strong absorption capacities. Additionally, they also possess large two photon absorption cross sections as well, making them outstanding candidates for designing PS-nanoconjugates with improved singlet oxygen quantum yield through two photon emission (TPE) as well as fluorescence resonance energy transfer (FRET) (Shen et al., 2016). They can also induce ^1^O_2_ and ROS production by transferring energy from the excited QD state to the ground state triplet oxygen, known as triplet energy transfer (TET). Moreover, they are much more photostable and biocompatible than organic dyes. Thus, quantum dots have been widely employed for bioimaging applications since their discovery (Freitas et al., 2020). Additionally, quantum dots combine several favorable characteristics making then suitable for PDT applications. Their biocompatibility and intracellular delivery can be easily improved by surface modifications (Bakalova et al., 2004). Although the concept of employing quantum dots as photosensitizers is rather nascent, few promising results have been obtained using carbon dots, graphene quantum dots and bimetallic dots.

### Fluorescence resonance energy transfer

The photoluminescence applications of QDs are usually governed by Fluorescence resonance energy transfer (FRET) and charge transfer interactions. These processes play crucial roles in quantum dots chemistry and applications. These photophysical processes can depend on several factors such as the overlap between acceptor absorbance and donor emission spectra, the number and orientation of acceptor around QD, the donor - acceptor distance, and the size and shape of the QD (Ji et al., 2016). FRET involves coupling of donor and acceptor dipoles. It can be explained as the non-radiative transfer of photoexcited donor energy to an adjacent ground state acceptor. When a donor group is excited by a photon, it relaxes back to the lowest level of the excited singlet state in accordance with Kasha’s rule. When an acceptor is present in close vicinity, the energy released during the relaxation of the donor to the ground state, may simultaneously excite the acceptor. This non radiative energy exchange is known as resonance. If the acceptor is also fluorescent, FRET serves to enhance the acceptor fluorescence. However, in case of a non-fluorescent acceptor, FRET results in quenching of donor fluorescence. Charge transfer can occur between any two donor acceptor molecules, not necessarily between fluorescent molecules. It involves the transfer of electrons or holes between the donor acceptor pair. Since these interactions vary as a function of donor acceptor distance, they find wide applications in sensing. Recently, quantum dot FRET has also been applied to enhance PDT action. QDs can be functionalized to enhance water solubility as well as conjugated with conventional photosensitizers and targeting molecules for improved therapeutic effect. In this regard, QDs can be used as effective drug carries for traditional PS to combat solubility and aggregation issues. Moreover, QDs can enhance the fluorescence emission of the photosensitizer drug by FRET interactions by several folds (Sewid et al., 2022). Suitable QD-PS conjugates are synthesized such that the QD emission overlaps with the PS excitation, thereby allowing the excitation of PS at lower wavelengths, as well as enhanced fluorescence emission by the PS.

### Prooxidant and antioxidant properties of QDs

A peculiar property characteristic of some quantum dots is the crossover from antioxidant to prooxidant nature upon laser irradiation. This was first depicted *in vitro* by Christensen et al. using bimetallic and carbon dots. They reported that the quantum dots inhibited oxidation of the radical probes used in the study, however, upon irradiation with blue light, they catalyzed the oxidation process. The generation of ^1^O_2_ was significantly enhanced in D_2_O solution. Thus, the quantum dots acted as oxygen scavengers in the dark, however upon irradiation with blue light, they induced the production of singlet oxygen. The crossover from antioxidant to prooxidant activity unlocked new avenues for QDs, particularly biocompatible carbon based QDs for photodynamic action (Christensen et al., 2011). Similar results were also reported by Chong et al. using graphene quantum dots. They concluded that in the absence of light, graphene quantum dots protected cells from oxidative damage by scavenging free radicals. However, upon irradiation with blue lights, they imposed significant cellular toxicity by generating reactive oxygen species (Chong et al., 2016). Another recent study using Au gold carbon nano dots (Au/NC) was reported by Zhao et al. The Au/NC prepared exhibited superoxide dismutase (SOD) like activity and depleted reactive oxygen species and free radicals in dark. However, on irradiation with visible light, the same Au/NC enhanced the generation of HO·, O_2_^−^, ^1^O_2_ and catalyzed the oxidation reactions (Zhao et al., 2021). Thus, smart quantum dots can be designed to induce cell protective and cytotoxic activities, which can be controlled by a light switch.

### Cell penetration mechanism

**FIGURE 6:**
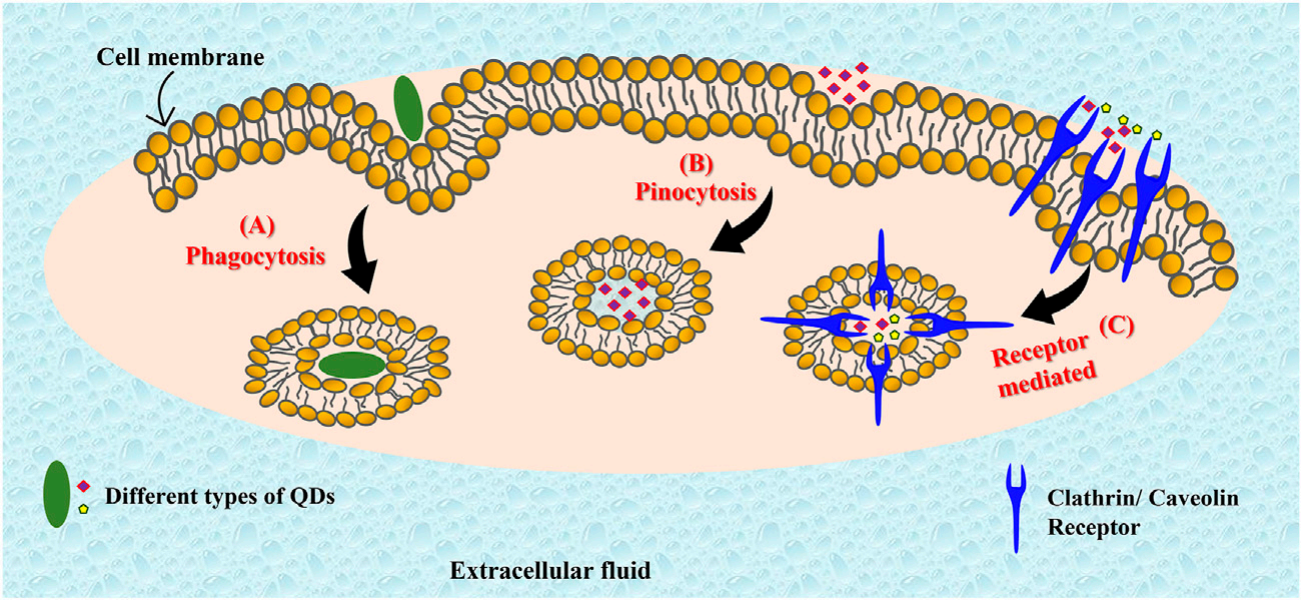
Different pathways of cell penetration that can be followed by quantum dots. Quantum dots can follow phagocytosis **(A)**, pinocytosis **(B)**, or receptor mediated pathways **(C)** for cell penetration.

With the tremendous potential for bioimaging and PDT, the understanding of cellular uptake mechanism and differential cellular localization of the quantum dots becomes pertinent. The cell membrane maintains the structural integrity of the cell. It has hydrophobic composition of phospholipid bilayers with embedded proteins. It acts as a permeable barrier allowing the passage of selected molecules inside the cell through active or passive transport. Passive transport follows a concentration gradient and is ATP (energy) independent. It is commonly employed by diffusion of gases such as oxygen, carbon dioxide and by hydrophobic molecules. Active transport involves the expenditure of energy in the form of ATP, and occurs against the concentration gradient, for example, endocytosis. It is commonly employed by polar/charged molecules that cannot pass through the hydrophobic cell membrane barrier. During this process, the cell engulfs the extracellular molecule inside by invagination (folding) of the cell membrane. The engulfed molecules are then brought inside the cell by slicing off cell membrane to form vesicles. Endocytosis can be further classified into three categories, pinocytosis, phagocytosis, and receptor mediated cytosis (Figure 6). Phagocytosis (cell eating) is a process of engulfing or ingesting of large particles (≥0.5 μm). Pinocytosis (cell drinking) involves the ingestion of liquid droplets of extracellular fluids with the dissolved molecules. (Foroozandeh and Aziz, 2018). The mechanism of cellular uptake adopted by the nanoparticle depends on their size, composition, surface modifications and charges. Many studies have reported the localization of various quantum dots in different cellular organelles in different cell lines. A detailed study by Xiao et al*.* described the cellular uptake of quantum dots (CdSe/ZnS) with -COOH coating, PEG, and amine derivatized PEG by MCF7 and MCF 10A cells without any specific surface functionalization on the respective QDs. They reported that only the -COOH coated QDs localized intracellularly in lysosomes in both the cell lines *via* clathrin-mediated endocytosis, while no detectable uptake was noted with the other two. The percentage of cellular uptake was greater in the cancerous cells (MCF 7) (Xiao et al., 2010). Similar results have also been reported by Liu et al. in their study with the bimetallic quantum dots (Cd/Se with ZnS shell) coated with mercaptoethylamine hydrochloride (QD-MEA) on Hela cells (Liu et al., 2021). The *in vitro* findings reported the clathrin-mediated, as well as actin and microtubule-dependent internalization of the QDs in the serum containing growth medium. However, in serum free media, the cellular uptake followed caveolae-mediated endocytosis and macropinocytosis, indicating the crucial role played by serum in the cellular uptake process. Furthermore, the QDs localized primarily in lysosomes, however some concentration could also be traced in mitochondria and endoplasmic reticulum. They also reported that the exocytosis of the internalized QDs was also rapid, however, only 40% was discharged. Metallic QDs often have limited scope for biological applications because of the severe toxicity risk imposed due to the high metal content. On the other hand, graphene quantum dots (GQDs) are often reported to be nontoxic and biocompatible for medical and therapeutic applications. GQDs are known to localize in the nucleus as well as the cytoplasm. Kumawat et al. have reported microwave synthesized, green GQDs that were shown to penetrate cell via caveolae and clathrin-mediated endocytosis and internalized with the cell nuclei without any capping agents (Kumawat et al., 2017). No cytotoxicity was observed up to 24 h of incubation. However, another study reported by Kersting et al. suggested localization of GQDs in late endosomes and lysosomes in MCF7, MDA-MB-231, and MCF 10A cells (Kersting et al., 2019). Aminated GQDs (AG-QDs) are also known to internalize within the cell nucleus of rat alveolar macrophages via caveolae-mediated endocytosis (Xu et al., 2018). Furthermore, the AG-QDs could also induce significant oxidative damage to DNA at higher concentrations. The easy uptake and internalization of QDs within the cell allows effective biomedical and bioimaging applications. Thus, most quantum dots follow endocytosis mechanisms for cell penetration and preferentially accumulate in the cytoplasm. However, they can be made to target other cellular organelles as well. Moreover, carbon based quantum dots are usually well tolerated and pose very little toxicity risk at therapeutic concentrations.

### Toxicity of quantum dots

The interest in quantum dots for biomedical applications has recently gained momentum, and detailed pharmacokinetics and pharmacodynamics pathways have not been detailed yet. Consequently, the use of heavy metal QDs for biomedical applications has been heavily disputed, however, the *in vitro* studies can provide significant insights for the development of biocompatible and nontoxic QDs. The toxicity related to heavy metal QDs is often attributed to leaching of the metal (eg Cd^2+^ from CdSe) and the subsequent cellular damage. Quantitative long term studies on the biodistribution of Cd based QDs suggest that the accumulation of Cd in kidneys and liver can reach up to 10 and 40% respectively as compared to the initial dosage (Yang et al., 2007). The biocompatibility of such QDs has been improved by encapsulation of the QDs within nontoxic coatings (amphiphilic polymers, ZnS shells), however, prolonged circulation often results in leaching of the naked QDs from the shell leading to cytotoxicity. Since the encapsulation usually occurs via weak interactions, the conjugates are susceptible to degradation and coagulation leading to cytotoxic effects (Smith et al., 2008). Nevertheless, carbon based quantum dots have proven to be less toxic and biocompatible (Wang et al., 2016). Most current studies indicate that carbon based quantum dots pose no significant toxicity to human cells *in vitro*. As compared to metallic quantum dots, carbon dots have much greater values of EC_50_ indicating safety and biocompatibility (Sun et al., 2020). Several studies indicate low to no toxicity of carbon based quantum dots, encouraging their development for biomedical and therapeutic modalities (Chan et al., 2021). Furthermore, uncharged PEGylated carbon dots have been found to be the most compatible QDs with least cytotoxicity *in vitro* (Havrdova et al., 2016). Overall, majority of the *in vitro* and *in vivo* studies on carbon and graphene quantum dots indicate low toxicity and excellent tolerance and biocompatibility. However, detailed pharmacokinetics and pharmacodynamics are warranted to establish their successful clinical applications.

## Quantum dots as photosensitizers in PDT

The application of semiconducting quantum dots for photodynamic therapy is an emerging field in its nascent stages. The preliminary results obtained so far are significant and promising and warrant further investigations for the complete development of semiconducting quantum dots as PDT agents. Most of the studies available in literature have been performed using bimetallic QDs, carbon QDs and graphene QDs. However, the pertinency of metallic QDs is limited due to the toxicity associated with heavy metals. Nevertheless, carbon and graphene based quantum dots are being increasingly explored for possible clinical applications. Few significant studies reported in literature using bimetallic, carbon based, and graphene based QDs are briefly discussed in the following sections.

### Bimetallic quantum dots

Metallic quantum dots including metal organic framework nano composites find extensive applications for bioimaging and energy storage devices (Huang et al., 2021; Li et al., 2021; Liu et al., 2022). The application of bimetallic quantum dots in PDT was initially proposed by Samia et al. using CdSe QDs linked to a photosensitizer (Pc4). The conjugated PS could be excited at 488 nm due to FRET interactions between the QD and the PS in the conjugate, while the fluorescence emission was observed at 680 nm (Samia et al., 2003). Their studies also predicted the capability of CdSe QDs to independently generate singlet oxygen as well, without the intervention of the classical photosensitizer (Pc4), although with a low quantum yield of only 5%. The decay emission of ^1^O_2_ was observed at 1,270 nm and was attributed to triplet energy transfer (TET) between the photoexcited QDs and the ground state oxygen (^3^O_2_). The results by Samia et al. suggested opportunities for developing quantum dots based photosensitizers for enhanced PDT action (Bakalova et al., 2004). Consequently, several studies have been reported on the usage of metallic quantum dots (especially with CdSe core) for PDT applications. Streptavidin functionalized CdSe-ZnS quantum dots conjugated to biotinylated pDNA also lead to DNA damage via reactive oxygen intermediates on photosensitization (Biju et al., 2010). To avoid the drawbacks associated with metallic quantum dots (such as metal leaching), some biocompatible modifications have also been suggested in literature. A noteworthy study was reported by Qi et al., by coating the metallic CdSe/ZnS QD with amphiphilic polymeric micelles. The resulting QDs were conjugated with a water soluble porphyrin (TrisMPyP-COOH) by covalent interactions. The conjugated QD-porphyrin displayed enhanced ^1^O_2_ generation capabilities via FRET and two photon excitations indicating the prospective bioimaging and PDT applications (Qi et al., 2011). Another interesting study was reported by Yan et al. describing the synthesis and applications of CdSe quantum dot-aza-BODIPY conjugate coated with folic acid and polyethylene glycol for theranostic applications via excitation at 635 nm. Efficient FRET from the QD to the aza dye resulted in enhanced fluorescence at 750 nm. Furthermore, the study also demonstrated the ability of the QD conjugate to generate ROS and induce phototoxicity to Hela cells on irradiation at 635 nm. The conjugate resulted in enhanced phototoxicity mediated by ROS generation. Interestingly, the study also suggested that the CdSe quantum dot-aza-BODIPY conjugate preferentially localized in cancer cells (Hela) while negligible localization was observed in normal cells, indicating high therapeutic efficacy with minimum side effects (Yan et al., 2016). Photosensitizing action of quantum dots containing CdSe core and ZnS shell has also been investigated against pancreatic cancer cells *in vitro*. The cells (SW 1990) were treated with different concentrations of the QDs followed by laser irradiation at 365 nm leading to apoptosis (He et al., 2016). More recently, Mn doped CuInSe quantum dots with a ZnS passivation layer (MnCuInSe/ZnS) have also been investigated for PDT and bioimaging applications via irradiation at 671 nm. The QDs could induce significant phototoxicity against HeLa, HepG2, and B16 cells, and were also found to be potent MRI contrasting agents (Irmania et al., 2022) Similar results indicating the theranostic ability of metallic quantum dots have also been reported by Li et al. using NIR-II-emitting and magnetic CuInSe_2_@ZnS:Mn QDs (Li et al., 2022c). Metallic quantum dots have also been explored for antibacterial photodynamic therapy (Gvozdev et al., 2018; Paul et al., 2022). Despite the success of metallic QDs for enhanced PDT applications, their wide usage has been restricted due to the potential toxicity posed by heavy metals such as Cd. However, carbon based quantum dots (carbon dots and graphene quantum dots) provide a safer and effective alternative.

### Carbon quantum dots

Carbon dots are photoluminescent, zero dimensional, carbon based nanomaterials with excellent light harvesting and electron transfer properties. They serve several advantages over metallic quantum dots, the most important being their biocompatibility and non-toxicity. In addition, they can be easily synthesized and exhibit high water dispersibility, as well as nonblinking fluorescence properties. Structurally, CDs possess an internal sp^2^ core while their surface is rich in -NH_2_, -COOH, -OH groups which allow for easy surface modifications. Also, doping of CDs with metals and heteroatoms can drastically alter the physical and optical properties of the CDs to cater to specific requirements (Li X. et al., 2022). They find extensive applications in fluorescence sensing (Yoo et al., 2019), photocatalysis (Sadjadi, 2021), electrochemistry (Hassanvand et al., 2021), and photovoltaics (Hutton et al., 2016; Essner and Baker, 2017). CDs have also been evaluated for their antibacterial photodynamic activity (Nie et al., 2020). Quite recently, CDs are also being explored for bioimaging and photodynamic therapy for cancer, mostly as theranostic agents. One of the earlier reports was the study by Huang *et al.* They reported the synthesis of multifunctional chlorin e6-conjugated PEG modified carbon dots (C-dots-Ce6) as theranostic agent for enhanced PDT and photosensitizer fluorescence detection (PFD). The prepared CD-PS conjugate exhibited a 35 fold increase in fluorescence intensity (at ∼668 nm) as compared to free chlorin e6 on excitation at 430 nm. This was attributed to the indirect excitation of Ce6 by CDs through fluorescence energy transfer (FRET). The PS-CD conjugate localized in the cytoplasm of gastric cancer cells, and the exposure to C-dots-Ce6 for 24 h followed by laser irradiation induced a concentration-dependent cytotoxicity. Moreover, the CDs were also shown to be effective for simultaneous enhanced fluorescence imaging and PDT of gastric tumor *in vivo* (Huang et al., 2012). Based on the same mechanism of FRET, Mg/N double-doped carbon dots with a high quantum yield were also reported by Yang et al. (Yang et al., 2016). They used 1,2-ethanediamine as the surface passivation agent as well as linkers for connecting the Mg/N doped CD with chlorin. The short distance between the CD and the PS resulted in enhanced FRET and a high quantum yield of 84.6%. The CD-chlorin conjugate displayed a strong fluorescence at 663 nm, ten times stronger than that of free chlorin, due to FRET interactions. Consequently, the CD–Ce6 system resulted in improved anticancer activity against HepG2 cancer cells as compared to PDT with chlorin alone. Apart from chlorin, CDs functionalized with riboflavin have also been reported for PDT for HeLa and melanoma cells with fivefold increased efficacy (Chowdhury et al., 2019).

Another interesting study using CD for targeting hypoxia in tumors using PDT was reported by Zheng et al. Hypoxia is a common characteristic in tumors that often result in resistance against PDT. Zheng et al. developed a carbon nitride doped carbon dot to photo catalyze the water splitting reaction to generate oxygen *in vivo* upon irradiation with 630 nm laser. This carbon dot was then conjugated with the PEGylated protoporphyrin PS for PDT applications. The conjugate was successful in downregulating hypoxia related proteins and improved the therapeutic effect of PDT (Zheng et al., 2016). Jia et al. reported the synthesis, bioimaging and PDT applications of CD from the powder of *Hypocrella bambusae*, a parasitic bamboo commonly used in Chinese traditional medicine (Jia et al., 2018). Selenium doped CDs were shown to selectively bind to RNA and damage the nuclear membrane by ROS generation (Xu et al., 2020). There are ample reports in literature suggesting the doping of CD with heteroatoms or metals such as N, P (Zhao et al., 2019), Cu (Wang et al., 2019), S (Li et al., 2020b), F/N (Wu et al., 2022), Hf (Su et al., 2020b), Sn (Hu et al., 2021) with enhanced bioimaging and PDT applications.

### Graphene quantum dots

Carbon dots are minute quasi spherical carbon nanoparticles, while graphene quantum dots (GQD) are small fragments of graphene with electron transport confined in three dimensions (Tian et al., 2018). The excitons in graphene have infinite Bohr radius and consequently GQDs exhibit appreciable luminescence properties. The band gap and (hence the luminescence) in GQDs can be fine-tuned by varying the size, and surface modifications and functionalization. Like CDs, GQDs are also environment friendly, have easy synthetic and functionalization routes and tunable opto—electrical properties (Ghosh et al., 2021). The potential and applications of GQDs are still being discovered, they find extensive applications in energy storage devices such as photovoltaics, light emitting diodes, and fuel cells. In biology, they are being actively explored for imaging (Fan et al., 2015; Xie et al., 2016; Younis et al., 2020) and PDT applications (Bacon et al., 2014). Earlier work on GQDs reported low cytotoxicity (Chong et al., 2014) with good cell penetration and uptake properties (endocytosis) (Su J. et al., 2020), indicating drug delivery (Iannazzo et al., 2017) and bioimaging applications. GQDs functionalized and conjugated with appropriate proteins and antibodies are commonly employed for biosensing (Fan et al., 2015; Xie et al., 2016; Suvarnaphaet and Pechprasarn, 2017; Mansuriya and Altintas, 2020; Tabish et al., 2021). Few examples from literature include GQD conjugated to antihuman immunoglobulin G antibody (mIgG) for sensing of immunoglobulin (Chen et al., 2018), glutathione functionalized GQD for sensing of ATP (Liu et al., 2013), N-functionalized GQD/Au nanoparticle/neuron-specific enolase antibodies (anti-NSE) bioconjugate for label free detection of small cell lung cancer (Kalkal et al., 2020), folic acid modifies/GQD/Au nanoparticle conjugate for ATP sensing (Zhang et al., 2019). Apart from imaging and sensing, GQDs are also gaining growing interest as photosensitizers for PDT. Earlier reports by Markovic et al. suggested the cytotoxicity of GQD against human glioma cells following irradiation at 470 nm by via reactive oxygen species like ^1^O_2_. The mechanism of cell death induced by photosensitized GQDs was proposed to follow autophagy and apoptosis (Markovic et al., 2012). Another study by the same group reported the cytotoxicity of photosensitized GQD against human rhabdomyosarcoma, human cervix carcinoma (HeLa) and fibroblasts post irradiation with blue light. However, the results were found to be less significant as compared to the positive control cisplatin (Marković et al., 2019). The application of GQD as a photosensitizer for PDT of MCF7 and B16F10 has also been reported by Ahirwar et al. Their study indicated low dark toxicity, but appreciable light toxicity by photosensitized GQD by irradiation with UV light *in vitro* (Ahirwar et al., 2020). Another interesting study was reported by Ju et al. proposing the fabrication of GQD to cater dual responsibilities, targeted drug delivery agent for doxorubicin, and a photosensitizer (Ju et al., 2019). They prepared nitrogen doped GQD loaded with doxorubicin and conjugated with charge reversal agent (3-Aminopropyl)triethoxysilane (APTES). Charge reversal agents and polymers are known to enhance the selectivity and localization of the drug within the cell nuclei. The N doped GQD/Dox/APTES conjugate presented excellent cytotoxicity against the highly proliferative MDA-MB-231 cells post irradiation using an LED with a wavelength of 622 nm and power output of 6.8 mW/cm^2^. Another noteworthy study was reported by Ge et al. They reported a highly water dispersible graphene quantum dot synthesized from polythiophene derivatives with an emission peak at 680 nm and broad absorption in the visible range (400–700 nm). The utility of the GQDs as fluorescence imaging agents were depicted by staining of Hela cells as well as *in vivo* studies on mice, with the GQDs staining only the cytoplasm and not the nucleus. *In vitro* cytotoxic studies indicated low cytotoxicity in the dark, but excellent light toxicity as compared to the conventional protoporphyrin IX (Ge et al., 2014). Interestingly, the GQDs prepared by Ge et al. afforded a substantial quantum yield of 1.3 due to multistate sensitization. Apart from cytotoxicity, GQDs have also been reported to upregulate host immunity proteins such as proinflammatory cytokines and T cells (Zhang et al., 2020). Graphitic carbon nitride quantum dots (g-CNQDs) have also been investigated for PDT by inducing significant oxidative stress in glioma cells following irradiation with blue light. The biocompatible g-CNQDs could induce apoptosis via decreased mitochondria membrane potential and activation of caspase 3/7 pathways (Yadav et al., 2022). Another recent study by Ramachandran et al. describes the synthesis and PDT applications of titanium dioxide nanoparticles and N-doped graphene quantum dots composites (N-GQDs/TiO2 NCs). The resulting nano composite could induce significant ROS generation along with cytotoxic effects against the highly aggressive MDA-MB-231 breast cancer cells following irradiation with NIR light (Ramachandran et al., 2022).

## Challenges and summary

**FIGURE 7:**
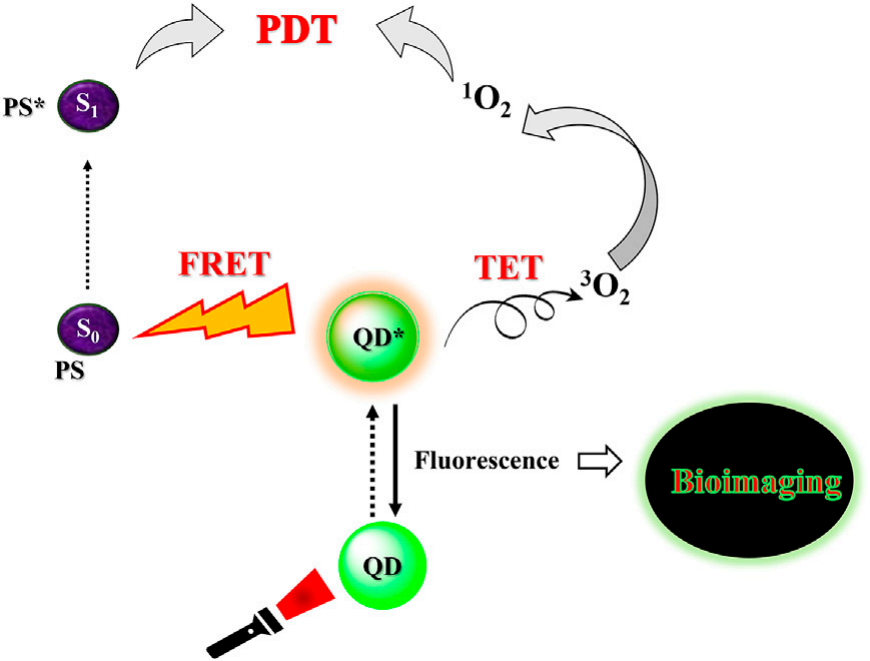
Simplistic representation of the applicability of quantum dots as photosensitizers for photodynamic therapy.

Quantum dots are the newest generation of nanomaterials that have garnered increasing attention due to their size tunable optical properties. They possess characteristic properties that can drastically transform cancer therapy and imaging. Most of the current research on QDs has been performed *in vitro*, their large two photon absorption cross section, photostability has motivated their applications for bioimaging of body organs as well as cellular organelles. Moreover, many QDs can generate singlet oxygen by triplet energy transfer (TET) and enhance the photoluminescence of classical photosensitizers via FRET (Figure 7). Carbon based QDs have low *in vitro* toxicity and their conjugation with classical PS can also assist in improving the aqueous dispersibility of organic PS. Moreover, the surface of quantum dots can be functionalized to assist in the attachment of specific antibodies and proteins to cause site specific localization of the PS, thereby combating phototoxicity commonly encountered with conventional photosensitizers. Therefore, the introduction of quantum dots to PDT can serve to eliminate most of the drawbacks experienced with current modalities. However, currently, there is very little data available on the pharmacokinetics and pharmacodynamics of quantum dots. The reports exploring the long term toxicity of quantum dots to humans as well as the environment are rather scarce in literature. The major drawback with metallic quantum dots is associated with toxicity imposed by heavy metals such as cadmium. Although, attempts have been made to overcome heavy metal toxicity by encapsulation in ZnS shells, however, few reports suggest some leching of the heavy metal after prolonged periods. Moreover, owing to their characteristic size distribution, nanoparticles can access cellular organelles which are impenetrable for metal ions. This may result in additional cytotoxicity as compared to free metals. Some preliminary studies suggest that exocytosis of quantum dots may not be as efficient as endocytosis, and they may accumulate in liver and kidneys leading to the risk of renal and hepatic toxicity. Thus, more preclinical data is required to understand the metabolism and excretion of quantum dots from human body. Each quantum dot formulation is unique, and thus current research needs to be focused on determining the lethal dose and inhibitory concentrations of quantum dots *in vivo* models. Furthermore, biocompatible metals such as copper and vanadium may be evaluated for the synthesis of less toxic quantum dots for biological applications. Carbon based quantum dots pose low toxicity risks and thus they can also be functionalized to improve specificity and therapeutic activity of conventional photosensitizers as well as metallic quantum dots by encapsulation. The conjugation of quantum dots with organic photosensitizers can be an excellent theranostic approach. Nevertheless, quantum dots have the potential to revolutionize photodynamic therapy and bioimaging; their detailed biochemical analysis is warranted for successful and safe clinical applications.
